# NLRP1 inhibits lung adenocarcinoma growth through mediating mitochondrial dysregulation in an inflammasome-independent manner

**DOI:** 10.1590/1414-431X2024e13885

**Published:** 2024-09-06

**Authors:** Chen-jing Lin, Guang-ang Tian, Wen-ya Zhao, Yi Tian, Yi-ru Liu, Dian-na Gu, Ling Tian

**Affiliations:** 1 Department of Central Laboratory, Shanghai Chest Hospital, Shanghai Jiao Tong University School of Medicine, Shanghai, China; 2 Department of Chemotherapy, The First Affiliated Hospital of Wenzhou Medical University, Wenzhou, Zhejiang, China

**Keywords:** NLRP1, Mitochondrial dysfunction, NF-κB signaling, Lung adenocarcinoma, Inflammasome

## Abstract

NLRP1, the first identified inflammasome-forming sensor, is thought to be involved in cancer, yet its definite function in lung adenocarcinoma (LUAD) remains unclear. Herein, we explored the expression and function of NLRP1 in LUAD. Decreased NLRP1 expression was identified in LUAD, which was associated with a poor prognosis. Overexpression of NLRP1 inhibited tumor growth *in vitro* and *in vivo*. Mechanically, this effect was observed regardless of inflammasome activation. Further studies revealed that overexpression of NLRP1 downregulated the phosphorylation of DRP1 and promoted mitochondrial fusion, which was mediated by inhibition of NF-κB activity. NF-κB agonist could neutralize the effect of NLRP1 on mitochondrial dynamics. In addition, LUAD sensitivity to cisplatin was enhanced by decreased mitochondrial fission resulting from up-regulated NLRP1. In conclusion, our findings demonstrated an unexpected role of NLRP1 in LUAD by modulating mitochondrial activities, which provides strong evidence for its potential in LUAD treatment.

## Introduction

Non-small cell lung cancer (NSCLC), the main histopathological type of lung cancer, is one of the most malignant cancers worldwide (1). Lung adenocarcinoma (LUAD), the most common NSCLC, occurs in smokers and non-smokers, regardless of age or gender. Currently, many therapeutic treatments are available, including surgery, chemotherapy, radiation, and immunotherapy, but the overall prognosis is still unsatisfactory (2). As lung is an organ that is constantly exposed to attacks from the external environment, it is thought that chronic inflammation may trigger carcinogenesis (3).

The innate immune system serves as the body's primary defense mechanism, which recognizes a variety of molecules, such as pathogen-associated molecular patterns, damage-associated molecular patterns, and disrupting cellular homeostasis to respond to host threats (4). The inflammasome is an important sentinel of the innate immune system, whose activation fundamentally alters the cellular state by regulating the balance between cell survival and death (5). Inflammasome hyper-activation contributes to the formation of an immunosuppressive microenvironment that promotes the progression of colitis-associated colorectal cancer (6). Inflammatory cell death induced by inflammasome activation plays a role in cytokine production and breast cancer pyroptosis (7). Essentially, inflammasome, inflammation, and cancer are an interrelated pathological triangle (8).

Nucleotide-binding oligomerization domain (NOD)-like receptor (NLR) family pyrin domain-containing 1 (NLRP1) was the first inflammasome-nucleating protein, identified at 2002 (9). NLRP1 protein is distinct from other NLRs due to the presence of additional domains, namely a function-to-find (FIIND) domain and a C-terminal caspase recruitment domain (CARD) (4). The FIIND of NLRP1 undergoing autoproteolytic cleavage was reported to activate caspase-1 and secrete interleukin (IL)-1β and IL-18 (10). NLRP1 is considered to be closely related to many inflammatory diseases. The activation of NLRP1 inflammasome triggered by lipopolysaccharide can induce oxidative stress, leading to reactive oxygen species (ROS) accumulation in the liver and the development of liver fibrosis (11). Inflammasome inhibition was reported to ameliorate ROS production (12). Equally, inhibition of ROS led to decreased inflammasome expression (13). It was found that NLRP1 might promote tumor growth through activation of inflammasomes and inhibition of apoptosis in metastatic melanoma (14). NLRP1 overexpression was observed to be correlated with tumorigenesis and proliferation in breast tumor (15). Lung epithelial cells usually express NLRP1 inflammasomes against pathogenic substances from the outside environment (16), but the definite function of NLRP1 in lung cancer remains unclear (17).

Herein, we explored the expression and function of NLRP1 in LUAD.

## Material and Methods

### Cell culture and treatment

Human LUAD cell lines PC-9 and H1299 derived from American Type Culture Collection (ATCC, USA) were cultured in RPMI-1640 (Gibco, USA) containing 10% fetal bovine serum (Wisent, Canada) and maintained in a 37°C incubator containing 5% CO_2_. The NLRP1 siRNAs were purchased from Ribobio (China), and NLRP1 overexpressed recombinant lentivirus was purchased from Genechem (China). For the activation of NF-κB signaling pathway, cells were treated with NF-κB activator 1 (MedChemexpress, USA) for 6 h.

### CCK-8 cell proliferation assay

A total of 5,000 cells were seeded onto 96-well plates and cultured. At the appropriate time (0, 24, 48, 72, and 96 h), 100 μL CCK-8 working solution (Adamas Life, China) was used to replace the old medium. After 1 h culture, Gen5 microplate reader (Bio Tek, USA) was used to detect the absorbance at 450 nm.

### Drug sensitivity analysis

The “oncoPredict” R package was employed to analyze the Genomics of Drug Sensitivity in Cancer (GDSC) database for evaluating the response to chemotherapy in high-expression and low-expression groups. Then, 10,000 cells with or without NLRP1 overexpression were seeded onto 96-well plates and cultured. After 48 h of treatment with cisplatin (MedChemexpress, USA), CCK-8 cell proliferation assay was performed according to the method described above.

### Flow cytometry assay

For cell cycle analysis, cells were resuspended in 100 μL PBS and incubated with PI (Multisciences Biotech, CCS012, China) at room temperature in the dark for 30 min. According to the manufacturer's instructions, Annexin V-APC/7-AAD apoptosis kit (Multisciences Biotech, AP105, China) was used for the apoptosis experiment. For ROS detection, Reactive Oxygen Species Assay kit (Beyotime, S0033S, China) was used following the instructions provided by the manufacturer. To detect mitochondrial membrane potential, the Enhanced Mitochondrial Membrane Potential Assay kit with JC-1 (Beyotime, C2003S) was used following the manufacturer's instructions. All analyses were conducted by BD FACS Canto^TM^ II (USA).

### Mitochondrial staining

The mitochondrial morphology of viable cells was observed using Mitotracker Red FM (Invitrogen, M22425, USA) according to the manufacturer's guidelines. Nuclei were counterstained with Hoechst 33342 (Thermo, 62249, USA). Images were captured by confocal laser scanning microscope (Zeiss LSM710, Germany) with an oil differential interference contrast objective.

### Western blot

Western blots were carried out according to standard protocols. The antibodies used are listed in Supplementary Table S1.

### ELISA

Cell supernatant was collected after 48 h seeding. ELISA was performed for IL-1β using Human Interleukin 1 Beta (IL-1β) ELISA Kit (Jianglaibio, JL13662, China) according to the manufacturer's instructions.

### RNA sequencing

Total RNA was isolated using TRIzol¯ Reagent (Sigma, USA). RNA-seq was performed by Biomarker Technologies (China).

### Immunohistochemistry

A tissue microarray was purchased from AiFang Biological (China). Informed consent was provided by all patients. Immunohistochemistry was carried out according to standard protocols. The intensity of staining was graded as follows: 0 (no staining), 1 (weak), 2 (moderate), or 3 (strong). Percentage scores were assigned as 1 (<25%), 2 (25-50%), 3 (51-75%), and 4 (>76%). Images were obtained under a light microscope (Grundium OCUS, Finland).

### Nude mouse xenograft studies

BALB/c nude mice (male, 4-6 weeks old, 16-18 g) were purchased from Shanghai JieSiJie Laboratory Animal (China) and maintained at the Experimental Animal Center of Shanghai Chest Hospital. Tumor volumes were monitored every 4 days and calculated as follows: volume (mm^3^) = length × width × width × 1/2. The animal research was approved by the Animal Care and Use Committee of Shanghai Chest Hospital.

### Statistical analysis

GraphPad Prism 5 software (USA) was used for statistical analysis. The data are reported as means±SD, and Student's *t*-test was utilized to determine the statistical significance.

## Results

### Decreased NLRP1 expression was correlated with poor prognosis in LUAD

**Figure 1:**
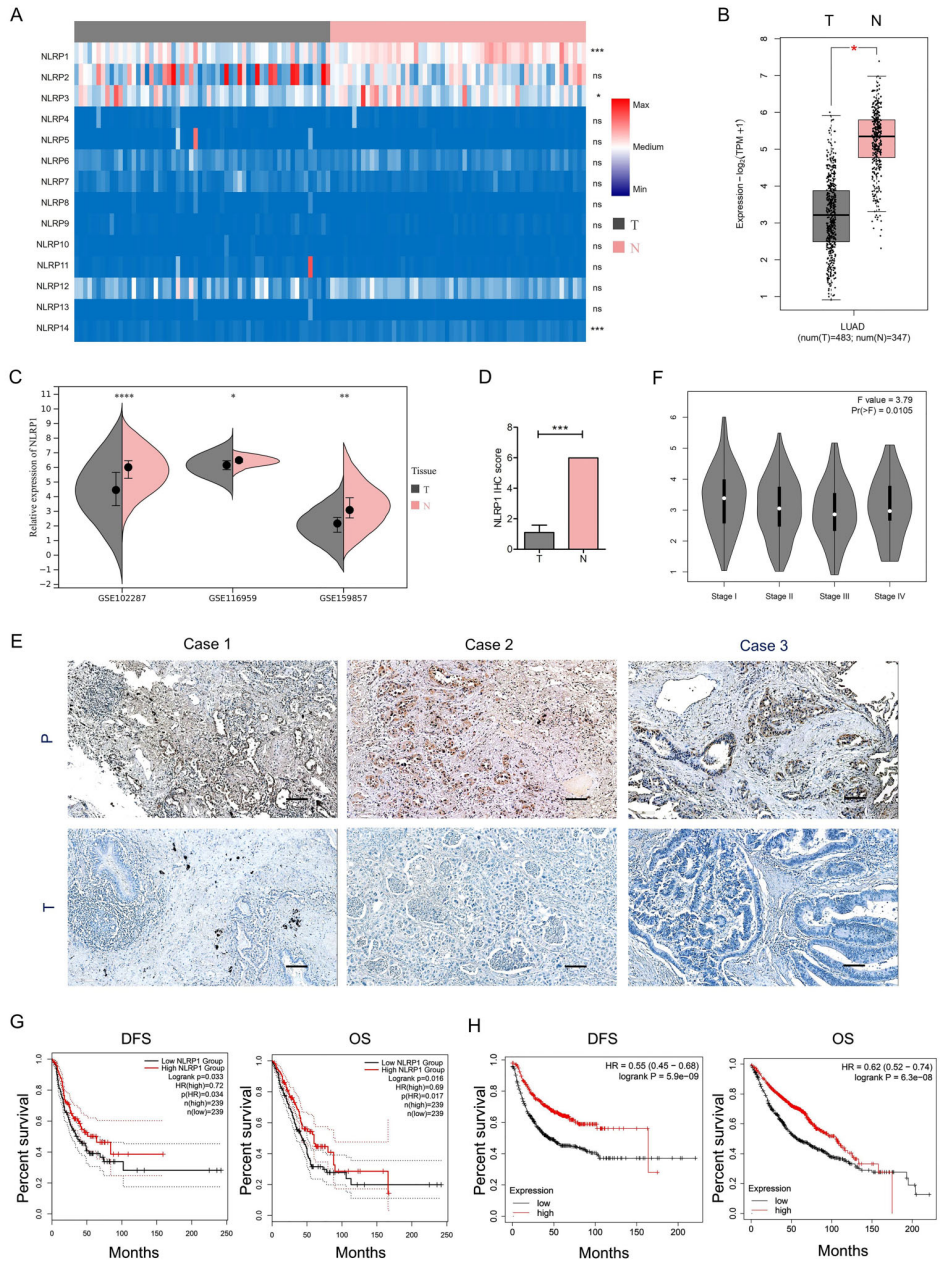
Decreased NLRP1 expression was correlated with poor clinical outcomes in lung adenocarcinoma (LUAD). **A**, Expression of NLRP family in LUAD and adjacent normal tissues. **B**, Expression level of NLRP1 in LUAD and adjacent non-tumor tissues from The Cancer Genome Atlas (TCGA) database was analyzed using GEPIA2. **C**, Expression analysis of NLRP1 in LUAD and adjacent normal tissues using 3 independent Gene Expression Omnibus (GEO) datasets. **D**, Immunostaining intensity score analysis of NLRP1 was analyzed using The Human Protein Atlas (THPA). **E**, Representative IHC images of the decreased NLRP1 expression in LUAD tissues compared with adjacent normal tissues. Scale bar, 100 μm. **F**, Association of NLRP1 expression with TNM stage was analyzed using GEPIA2. **G** and **H**, Prognostic value of NLRP1 in LUAD disease-free survival (DFS) and overall survival (OS) was analyzed using GEPIA2 (**G**) and K-M Plotter (**H**). T: Tumor tissue; N: Normal tissue; P: Paracancerous tissue. Data are reported as mean and SD. *P<0.05, **P<0.01, ***P<0.001, ****P<0.0001; Student's *t*-test.

To assess the expression of inflammasome related proteins in LUAD, The Cancer Genome Atlas (TCGA) database was utilized to detect the NLRP family expression. NLRP1 had the most significant change between paired cancer and adjacent tissue, which was significantly decreased in LUAD (Figure 1A and B). Similar expression trends were obtained from the analysis of multiple Gene Expression Omnibus (GEO) datasets (GSE102287, GSE116959, and GSE159857) (Figure 1C). Consistently, immunohistochemistry (IHC) staining also revealed significantly down-regulated expression of NLRP1 in cancer compared with adjacent tissue, using both The Human Protein Atlas (THPA) (Figure 1D) and our collected 3 paired LUAD tissues and adjacent tissue (Figure 1E). The prognostic value of NLRP1 in LUAD was assessed using the GEPIA2 and Kaplan-Meier plotter database. The results demonstrated a positive correlation between NLRP1 expression and patient outcomes, including clinical stages, disease-free survival (DFS), and overall survival (OS), based on median values (Figure 1F-H).

**Figure 2:**
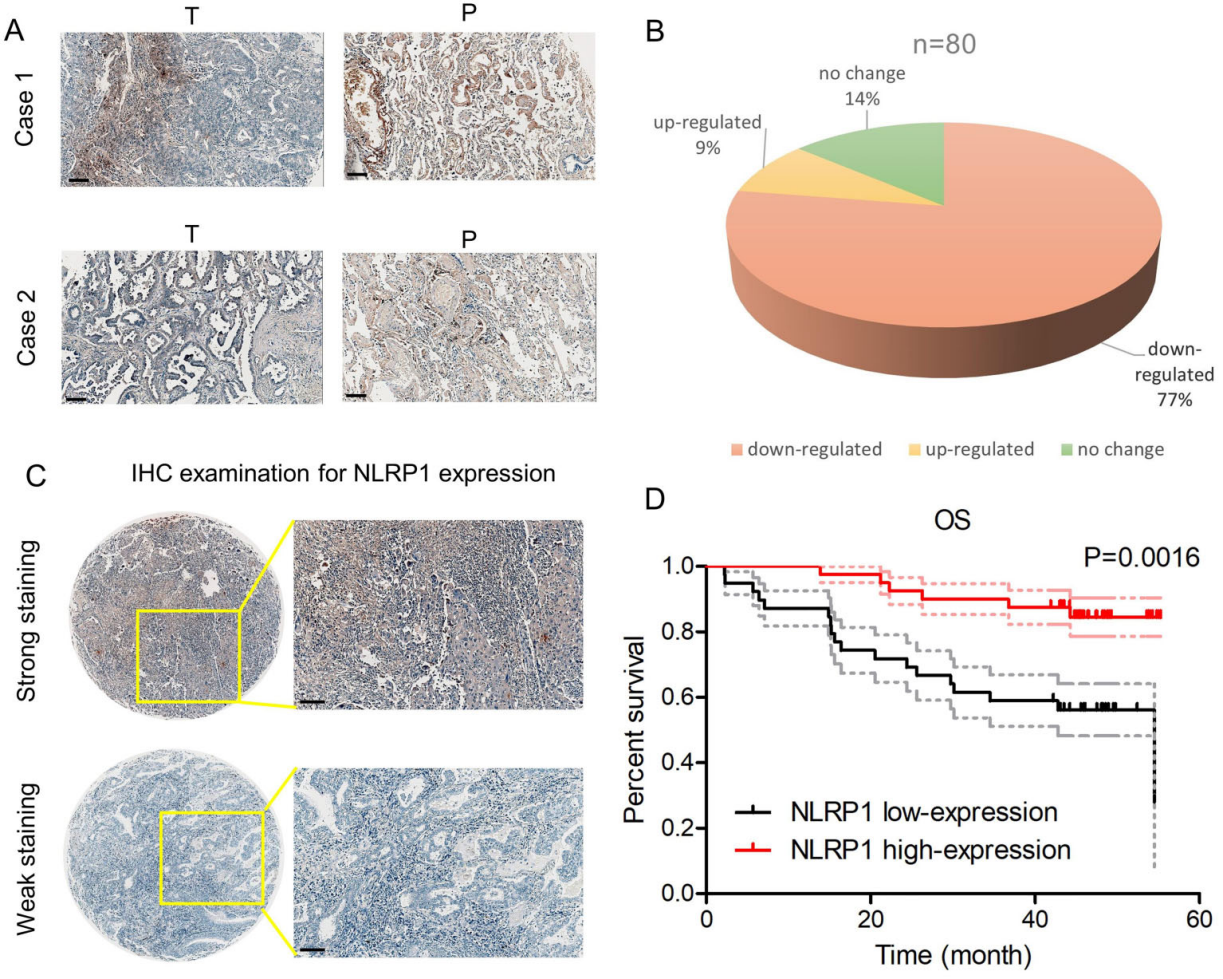
Decreased NLRP1 expression was correlated with poor clinical outcomes in lung adenocarcinoma (LUAD). **A** and **B**, Expression level of NLRP1 in LUAD and adjacent non-tumor tissues from tissue microarray. Scale bar, 100 μm. **C**, Representative images for strong staining and weak staining of NLRP1 in LUAD. Scale bar, 100 μm. **D**, Overall survival (OS) analysis of LUAD patients divided by NLRP1 expression. T: Tumor tissue; P: Paracancerous tissue.

IHC of NLRP1 was performed in 80 tissue samples, which were pathologically diagnosed as LUAD, to confirm this result. A significant decrease of NLRP1 expression was found in LUAD tissues among 77% of patients (Figure 2A and B). Representative images for NLRP1 in LUAD are shown in Figure 2C. In line with the aforementioned findings, patients with high NLRP1 expression had a better prognosis (Figure 2D).

However, although NLRP1 was also decreased in lung squamous cell carcinoma (LUSC), it did not have an effect on prognosis (Supplementary Figure S1). Collectively, these data indicated that the decreased NLRP1 expression was highly correlated with poor clinical outcomes in LUAD patients.

### NLRP1 overexpression inhibited LUAD growth *in vitro* and *in vivo*

**Figure 3:**
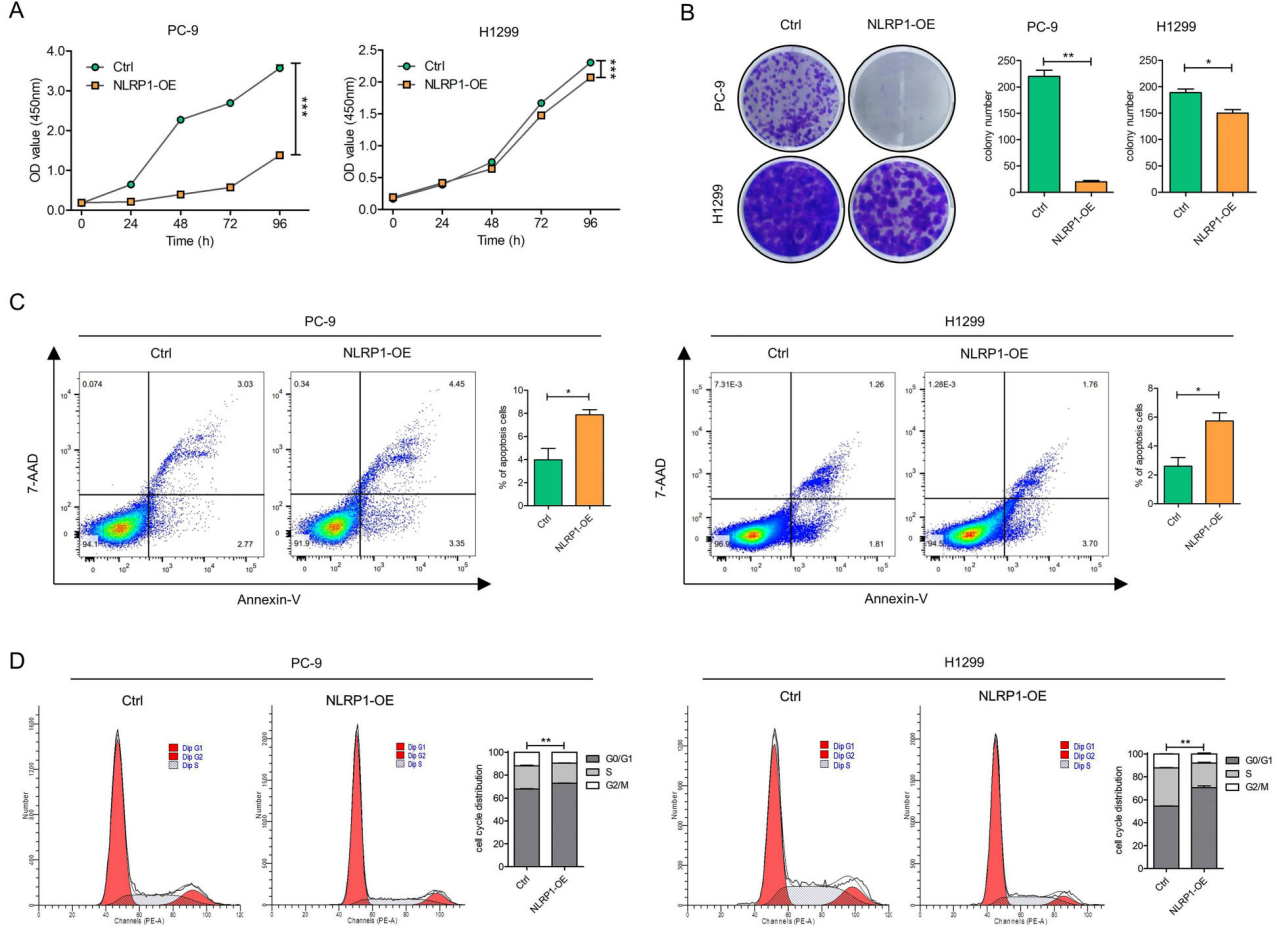
NLRP1 overexpression inhibited lung adenocarcinoma (LUAD) cell proliferation *in vitro.* **A**, Cell viability was determined by CCK8 assay in PC-9 and H1299 cells with stable overexpression (OE) of NLRP1. **B**, Colony formation assay was performed in PC-9 cells and H1299 cells with NLRP1 overexpression and control. **C**, Cell apoptosis of PC-9 and H1299 cells with stable overexpression of NLRP1 was assessed by flow cytometry assay with Annexin V/7-AAD double staining. **D**, Cell cycle arrest was determined for PC-9 cells and H1299 cells with NLRP1 overexpression and control. Data are reported as mean and SD. *P<0.05, **P<0.01, ***P<0.001; Student's *t*-test.

To further elucidate the biological functions of NLRP1 in LUAD, cells with up-regulated and down-regulated NLRP1 expression were generated (Supplementary Figure S2A and B). Cell proliferation rate was evaluated, and LUAD cells with overexpressed NLRP1 showed a significant decrease in proliferation capability (Figure 3A and B). Further, it was found that cancer cells with NLRP1 overexpression exhibited a much higher apoptosis rate (nearly doubled) (Figure 3C). Moreover, the cell cycle of NLRP1-overexpressing cancer cells might be arrested in G1 phase (Figure 3D).

**Figure 4:**
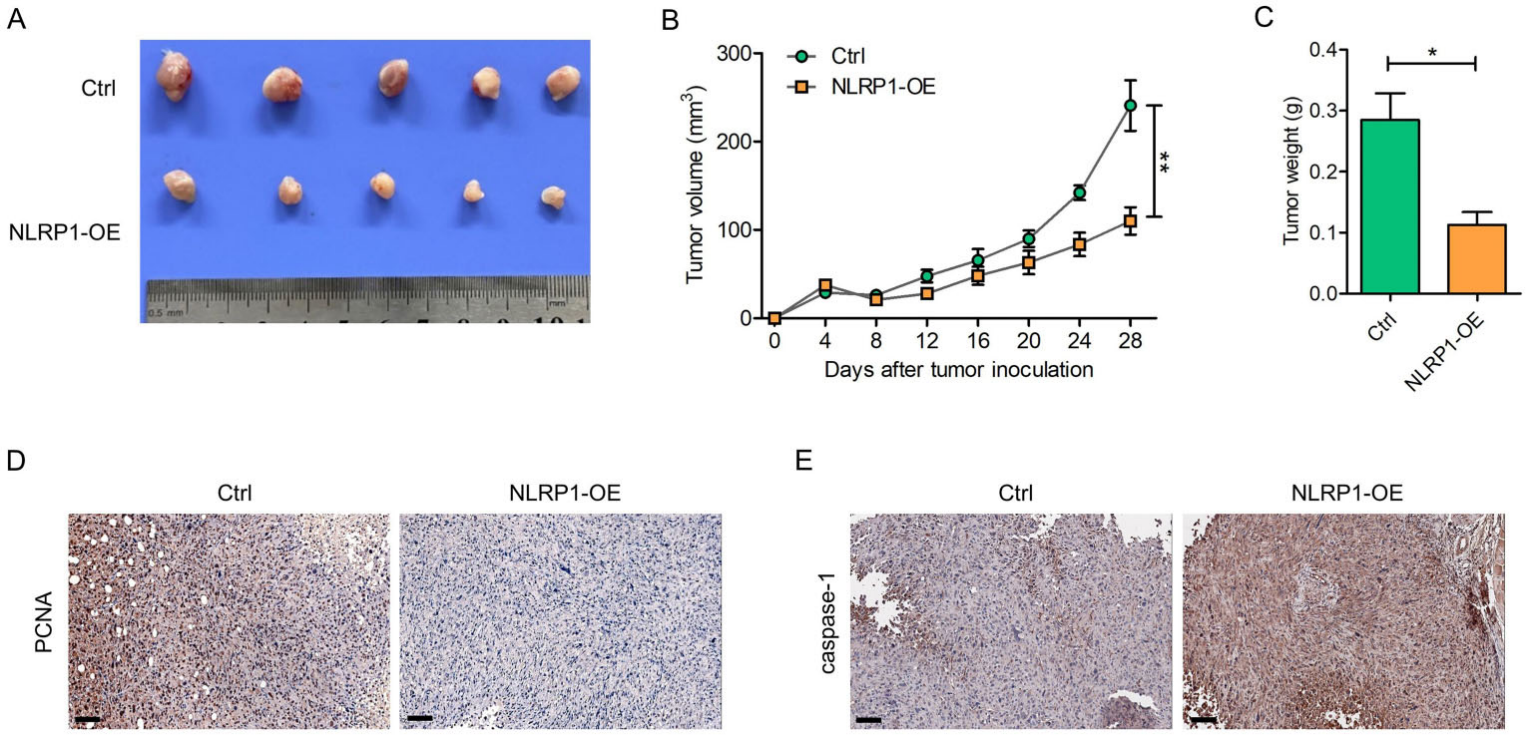
NLRP1 overexpression inhibited lung adenocarcinoma (LUAD) growth *in vivo*. **A**-**C**, Representative tumor images (**A**), growth curves (**B**), and weights (**C**) in BALB/c mice bearing PC-9 cells with or without NLRP1 overexpression (OE) (n=5). **D**, Representative IHC images of PCNA staining in BALB/c tumor-bearing mice. Scale bar, 100 μm. E, Representative IHC images of capase-1 staining in mice tumors. Scale bar, 100 μm. Data are reported as mean and SD. *P<0.05, **P<0.01; Student's *t*-test.

Nude mouse xenograft models were then established to compare their tumorigenesis capability. Remarkably, the NLRP1 overexpression group displayed a significantly reduced tumor burden (Figure 4A-C). Furthermore, IHC assay revealed a significant reduction in positive staining of the proliferating cell nuclear antigen (PCNA) in NLRP1 overexpression group (Figure 4D), whereas the expression of apoptosis-related protein capase-1 was increased (Figure 4E). Together, these results suggested that NLRP1 overexpression inhibited LUAD growth *in vitro* and *in vivo*.

### NLRP1 overexpression was independent of inflammasome activation in LUAD cells

**Figure 5:**
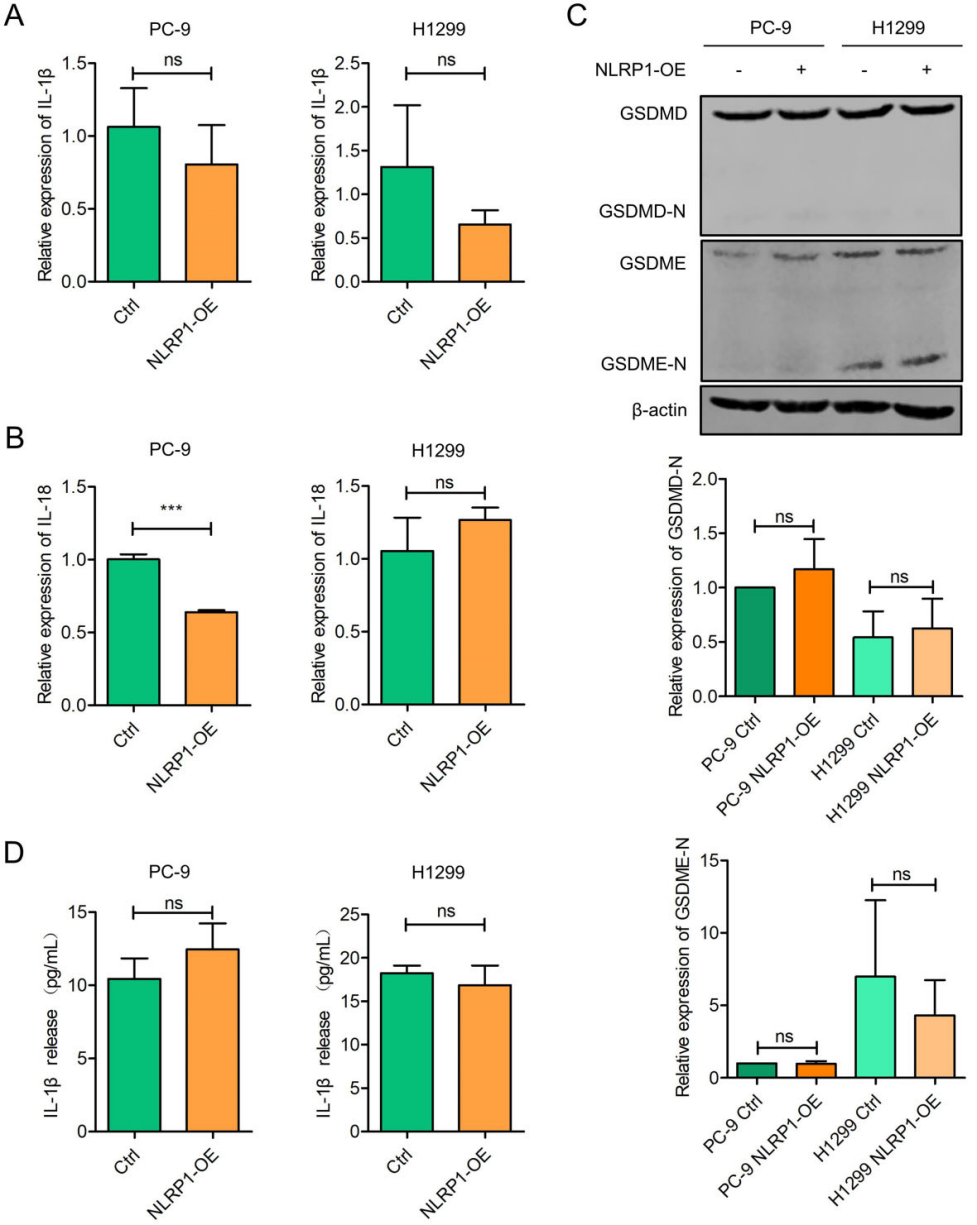
NLRP1 overexpression (OE) did not activate inflammasome in lung adenocarcinoma (LUAD) cells. **A**, RT-qPCR was performed to assess relative mRNA expression of interleukin (IL)-1β in NLRP1 stably-transfected LUAD cells. **B**, RT-qPCR was performed to assess relative mRNA expression of IL-18 in NLRP1 stably-transfected LUAD cells. **C**, Western blot was used to detect the expression levels of GSDMD and GSDME, and the quantification of the bands is shown in the lower part. **D**, Cell supernatant was harvested for ELISA detection of secreted IL-1β. Data are reported as mean and SD. ***P<0.001; Student's *t*-test. ns: no significance.

It is known that inflammasomes participate in the formation of a high-molecular-weight signaling platform for recruiting caspase-1 (18). Caspase-1 activated by the inflammasomes cleaves gasdermin (GSDM) family and generates N-terminal fragments, which form pores in the cytoplasmic membrane, resulting in pyroptotic cell death and leakage of cell contents, such as cytokines IL-1β and IL-18 (16). Accordingly, we examined the activation of inflammasomes. Despite the overexpression of NLRP1, no increase in mRNA expression of IL-1β and IL-18 was observed (Figure 5A and B). Western blots showed no trend of activation for either GSDMD or GSDME (Figure 5C). Consistent with the above results, there was no discernible increase in IL-1β secretion detected in the cell supernatant in NLRP1-overexpressing cells (Figure 5D). These results indicated that this mechanism was independent of inflammasome activation in LUAD cells.

### NLRP1 overexpression caused mitochondrial dysfunction in LUAD cells

**Figure 6:**
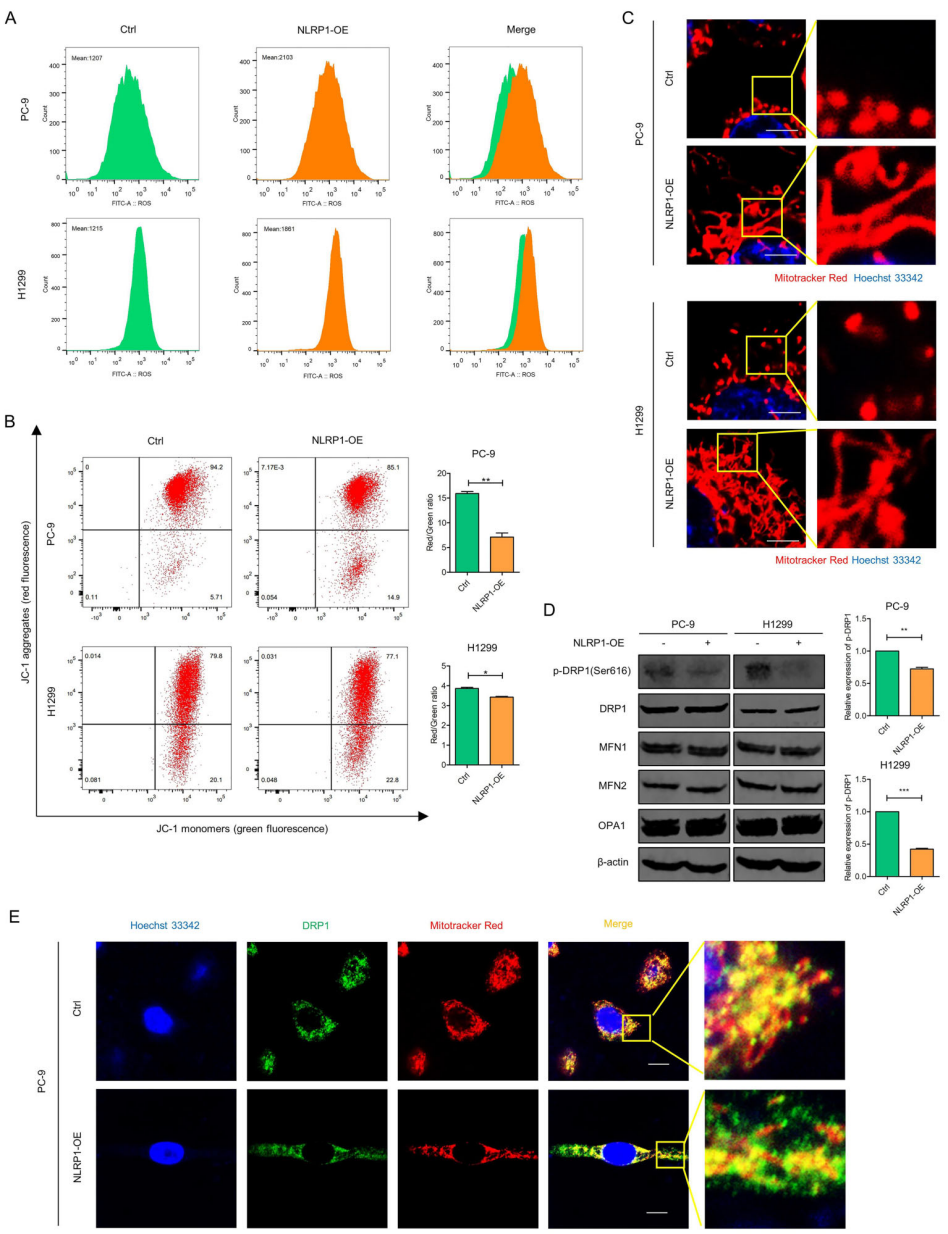
NLRP1 overexpression (OE) inhibited mitochondria function. **A**, Reactive oxygen species (ROS) levels were detected by flow cytometry assay with DCFH-DA fluorescent probe. **B**, Mitochondrial membrane potential was evaluated by flow cytometry analysis with JC-1 fluorescent probe. **C**, NLRP1-induced mitochondrial fusion in PC-9 and H1299 cells was detected by confocal microscopy. Scale bar, 20 μm. **D**, Expression levels of p-DRP1, DRP1, MFN1, MFN2, and OPA1 were analyzed by western blot, and the quantification of the bands is shown on the right. **E**, Co-localization of DRP1 with mitochondria was detected by confocal microscopy. Scale bar, 10 μm. Data are reported as mean and SD. *P<0.05, **P<0.01, ***P<0.001; Student's *t*-test.

Therefore, we turned to exploring mitochondrial function. It has been recognized that the reciprocal regulation between ROS and inflammasomes is a common pathological mechanism in various diseases. Not surprisingly, ROS was significantly raised in LUAD cells with NLRP1 overexpression (Figure 6A). Accordingly, the NLRP1-overexpressing group exhibited mitochondrial membrane potential (MMP) depolarization, as evidenced by the JC-1 fluorescent probe (Figure 6B). These results indicated that NLRP1 overexpression caused mitochondrial dysfunction in LUAD cells. Then, mitochondrial networking status was examined, and LUAD cells with NLRP1 overexpression exhibited a significant elongation of mitochondrial elements, displaying a network of mitochondria with a tubular morphology, while the predominant mitochondrial morphology in the control cells was characterized by fragmentation and short tubules (Figure 6C).

Given that lung cancer cells have a strong mitochondrial fission activity to promote tumor progression, it might be reasonable to infer that NLRP1 had the ability to inhibit tumor cell growth, possibly by promoting the transition of mitochondria from a fragmented state to a fused state. Thus, several key proteins involved in it were assessed. No significant variation was observed in the levels of pro-fission molecule DRP1 (dynamin-related protein 1) and pro-fusion molecules MFN1 (mitofusin 1), MFN2, and OPA1 (optic atrophy 1) between LUAD cells with or without NLRP1 overexpression, while the phosphorylation level of DRP1 at serine 616 (S616) was found to be significantly inhibited in NLRP1-overexpressing cells (Figure 6D). The recruitment of DRP1 to mitochondria was consistently restrained (Figure 6E). These findings collectively suggested that NLRP1 mediated mitochondrial dynamics and significantly influenced mitochondrial function in LUAD cells.

### NLRP1 overexpression reduced NF-κB activity, mediating mitochondria fusion

**Figure 7:**
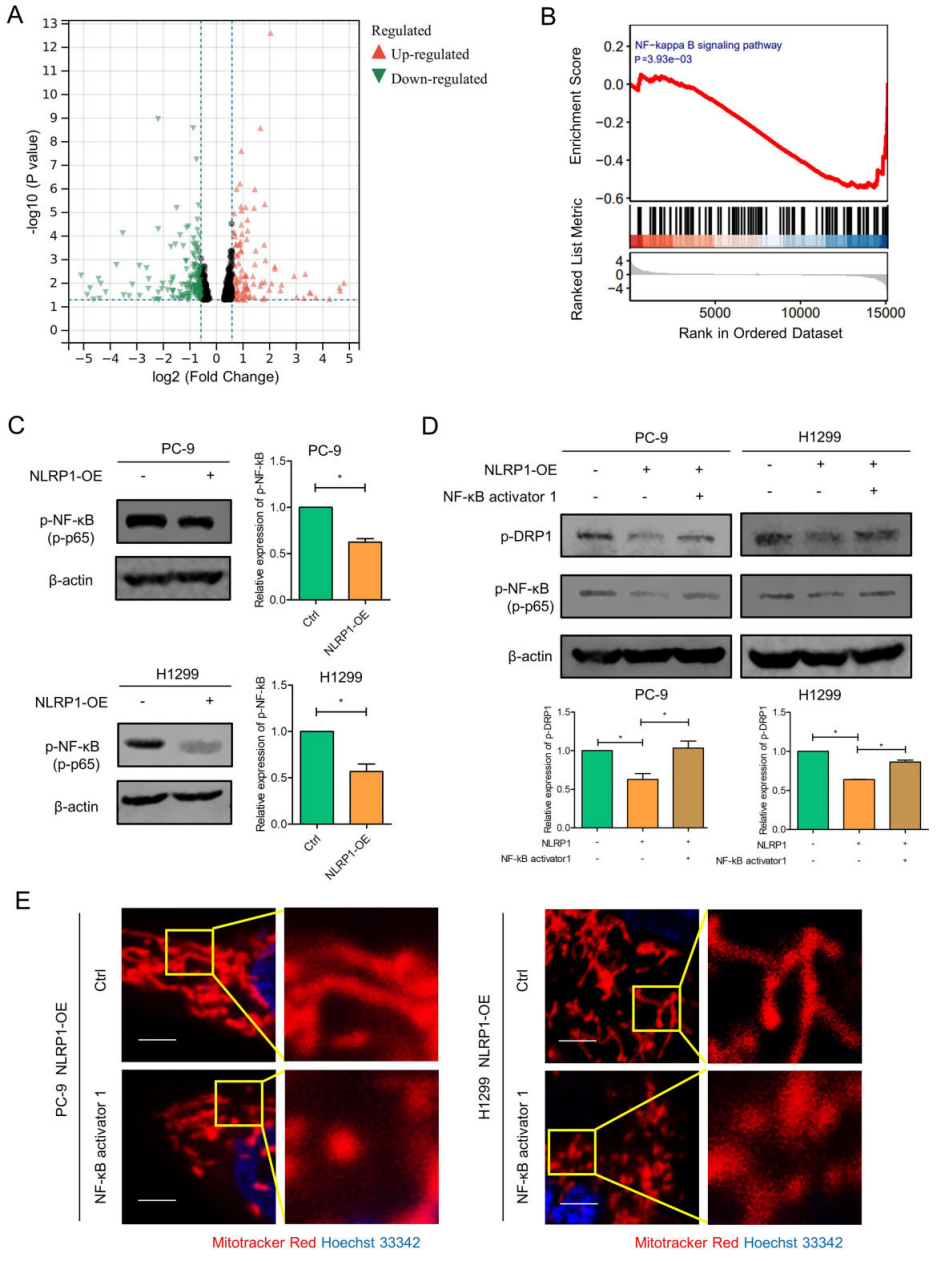
NLRP1 overexpression (OE) reduced NF-κB activity and mediated mitochondria fusion. **A**, DEseq2 analysis showed differentially expressed genes (DEGs) between control group and NLRP1 overexpression groups in PC-9 cells. **B**, Gene Set Enrichment Analysis (GSEA) revealed that NF-κB signaling was enriched in the control group but not in the NLRP1 overexpression group. **C**, NF-κB phosphorylation (p-p65) expression was detected by western blot in NLRP1 stable-transfected lung adenocarcinoma (LUAD) cells, and the quantification of the bands is shown on the right. **D**, NF-κB phosphorylation (p-p65) and DRP1 phosphorylation (Ser616) expression were detected by western blot, and the quantification of the bands is shown in the lower part. **E**, Mitochondrial morphology was detected by confocal microscopy in lung cancer cells treated with DMSO or NF-κB activator 1. Scale bar, 10 μm. Data are reported as mean and SD. *P<0.05; Student's *t*-test.

To further explore the molecular mechanism by which NLRP1 affected mitochondrial dynamics, RNA-seq was performed and analyzed. DEseq2 analysis showed that around 629 genes were significantly altered in NLRP1-overexpressing PC-9 cells (Figure 7A). The genes related to NF-κB (nuclear factor-κB) signaling were enriched in the control group but not in the NLRP1-overexpression group according to Gene Set Enrichment Analysis (GSEA) of DEGs (Figure 7B). Accordingly, the phosphorylation of NF-κB was detected, and reduced NF-κB activity was found in NLRP1 stable-transfected LUAD cells (Figure 7C).

Recently, it was found that targeting NF-κB/DRP1 could alter mitochondrial morphology and function in hippocampal neurons (19). Therefore, it would be reasonable to speculate that NF-κB might be involved in NLRP1-regulated mitochondrial fusion. NF-κB activator 1 was used to determine whether NF-κB was mediated in NLRP1-induced mitochondrial dynamic changes. It was found that the level of DRP1 phosphorylation in LUAD cells with restored NF-κB activity was similar to that in the control group (Figure 7D). Accordingly, restoration of NF-κB activity was observed to promote mitochondria fission, counteracting the pro-fusion effect of NLRP1 on mitochondria (Figure 7E). Together, these evidences indicated that NLRP1 overexpression could reduce NF-κB activity, subsequently mediating mitochondria fusion.

### NLRP1 enhanced LUAD sensitivity to cisplatin by inhibiting DRP1 activity

**Figure 8:**
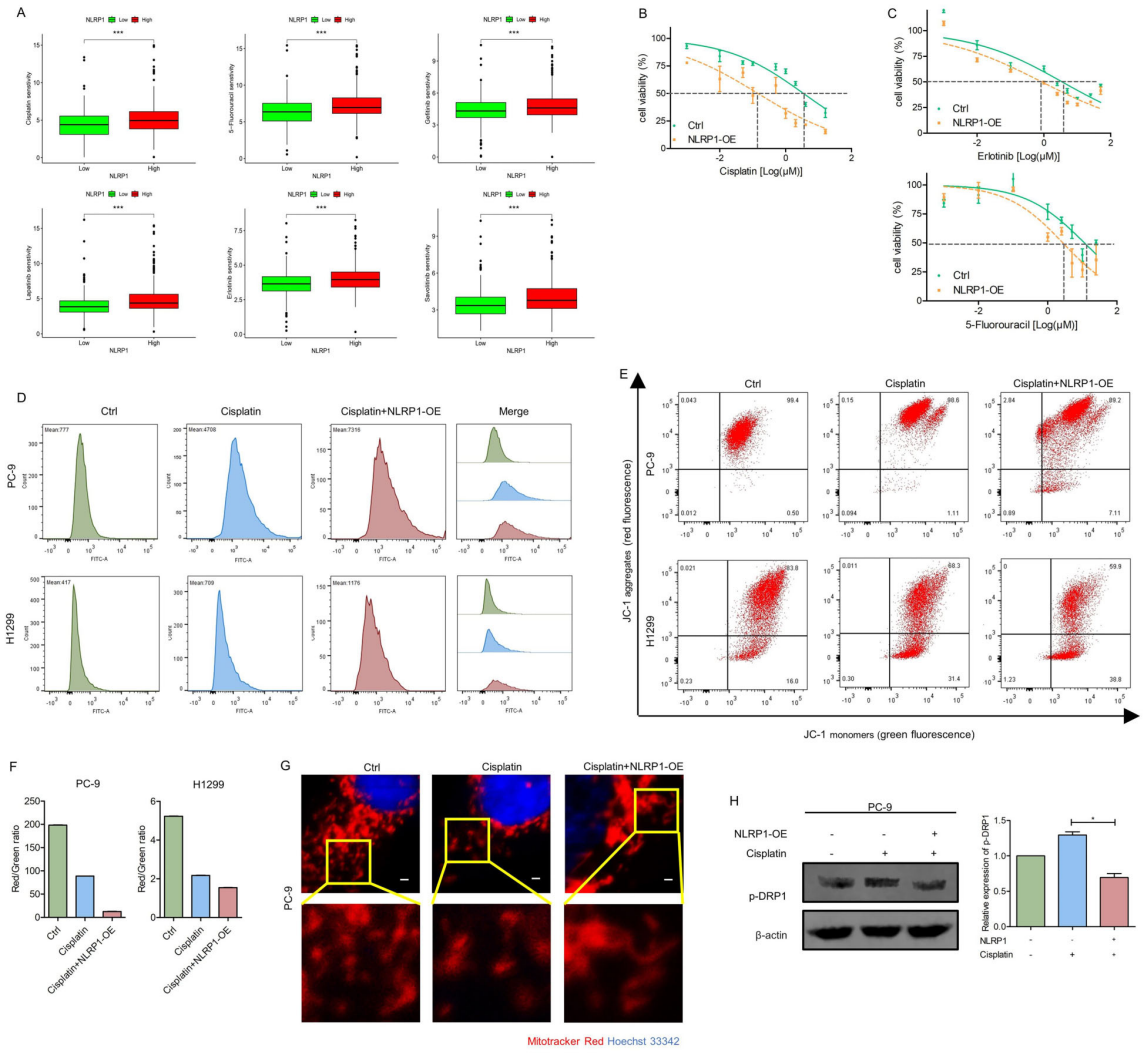
NLRP1 enhanced lung adenocarcinoma (LUAD) sensitivity to cisplatin by inhibiting DRP1 activity. **A**, Genomics of Drug Sensitivity in Cancer (GDSC) database was used to predict the drug sensitivity of NLRP1. **B**, NLRP1 overexpression (OE) enhanced cisplatin sensitivity according to CCK8 assay. **C** NLRP1 overexpression increased 5-fluorouracil and erlotinib sensitivity. **D**, Reactive oxygen species levels were detected by DCFH-DA fluorescent probe. **E** and **F**, Mitochondrial membrane potential was evaluated by flow cytometry assay with JC-1 fluorescent probe. **G**, Mitochondrial morphology was detected by confocal microscopy. Scale bar, 10 μm. **H**, The phosphorylation level of DRP1(Ser616) in control and NLRP1-overexpressing cancer cells after cisplatin treatment was detected by western blot, and the quantification of the bands is shown on the right. Data are reported as mean and SD. *P<0.05, ***P<0.001; Student's *t*-test.

The sensitivity of LUAD to chemotherapeutic drugs was assessed to further evaluate the potential value of NLRP1 in clinical treatment. Remarkably, patients with high NLRP1 expression were more sensitive not only to traditional chemotherapy drugs (such as cisplatin and 5-fluorouracil, etc.), but also to targeted drugs such as EGFR-TKIs (epidermal growth factor receptor-tyrosine kinase inhibitors such as gefitinib, lapatinib, and erlotinib, etc.) and MET inhibitors (such as savolitinib, etc.) (Figure 8A). CCK-8 cell proliferation assay was then performed to assess cell viability of PC-9 cells, and we found that NLRP1 not only enhanced LUAD sensitivity to cisplatin (Figure 8B), but also to 5-fluorouracil and erlotinib (Figure 8C).

Moreover, NLRP1 overexpression increased cisplatin-induced ROS release in LUAD cells (Figure 8D). The decrease in MMP was more pronounced when NLRP1 overexpression was combined with cisplatin (Figure 8E and F). As evidenced by mitochondrial morphology, mitochondrial fission was increased after cisplatin treatment, while the predominantly mitochondria morphology was reversed by NLRP1 overexpression (Figure 8G). Moreover, western blot showed that DRP1 phosphorylation was increased by cisplatin, while NLRP1 overexpression partly mitigated this effect (Figure 8H). Together, these results suggested that NLRP1 overexpression would contribute to sensitizing LUAD cells to cisplatin through inducing mitochondrial dysfunction.

## Discussion

As an important component of the innate immune response, inflammasomes seem to play diverse and conflicting roles in tumors with a significant context-dependency (8). It was reported that NLRP1 signaling is silenced to favor advanced skin tumor (20), while the expression of the NLRP1 inflammasome might promote prostate cancer through the production of proinflammatory cytokines, namely IL-1β and IL-18 (21). As lung is an inflammation-prone tissue because of its frequent exposure to external substances, NLRP1 is thought to play an important role in it (17). Recently, Shen et al. (22) reported that the low expression of NLRP1 might correlate with a poor prognosis in LUAD patients through bioinformatics data analysis. In this study, we also demonstrated that NLRP1 expression was significantly decreased in LUAD, and its downregulation promoted tumor progression. It seemed that the effect of tumor inhibition was more pronounced in NLRP1-overexpressing PC-9 cells compared to H1299 cells, which might be partially due to their different basal expression of NLRP1 (Supplementary Figure S2A). However, it should be noted that NLRP1 silencing in LUAD cells did not obviously affect cell proliferation (Supplementary Figure S2C), which indicated that the relatively low basal expression level of NLRP1 in these cells might contribute to their proliferation.

In this study, we investigated the underlying molecular mechanism and revealed that NLRP1 suppressed LUAD growth not through activating inflammasomes but rather by mediating mitochondrial dysregulation. NLRP1 usually detects damage-associated molecular patterns to form a multiprotein complex (18). Upon assembly of the inflammasome, caspase-1 undergoes self-activation and enzymatically processes proinflammatory cytokines from the IL-1 family, as well as members of the gasdermin family (16). Contrary to the belief that inflammasomes facilitate inflammation, NLRP3 overexpression attenuated AIM2 transcription to inhibit inflammasome activation (23). In an inflammasome-independent manner, NLRP3 has been shown to modulate energy metabolism, thereby enhancing the therapeutic effects of mesenchymal stem cells (24). Consistent with these findings, we proposed a novel role for the NLRP1 molecule in LUAD independent of inflammasome activation.

A subtle balance between ROS generation and scavenging can keep cancer cells within the tumorigenic range of ROS levels (25). Indeed, NLRP1 activation could be increased via ROS production, while ROS generation could also derive from NLRs inflammation activation (26). As mitochondria are the major source of intracellular ROS, both ROS production and inflammasome activation were suppressed when mitochondrial activity was dysregulated (27). Supposedly, there was a positive feedback loop between inflammasome activation and mitochondrial damage. As known, mitochondria are remarkably dynamic organelles that act as metabolic hubs and signaling platforms (28). Individual mitochondria adopt a variety of morphologies, including round, short or long tubules, and interconnected tubules (29), which are thought to be regulated by the post-translational modifications of the dynamin family of GTPase proteins (30). It was suggested that the phenotypes of lung cancer are associated with small and fragmented mitochondria, which generated more energy to support rapid proliferation and metastasis of cancer (31). In particular, inhibiting DRP1 has shown promising effects on reducing cell proliferation and promoting apoptosis, while increased DRP1 activity appears to contribute to tumor progression driven by oncogenic Ras (32). We proved for the first time the NLRP1-mediated mitochondrial dynamics of fusion and fission. Our data showed that NLRP1 overexpression reduced mitochondrial membrane potential and inhibited mitochondrial fission in lung cancer. Phosphorylation of DRP1 at S616 was decreased, and aggregation of DRP1 at mitochondrial sites was inhibited. Nevertheless, the regulatory mechanisms of mitochondria and their function in lung cancer cells need to be further explored.

NF-κB was identified as a crucial factor in regulating mitochondrial dynamics. Increased NF-kB activity and nitric oxide concentrations led to a notable shift of mitochondrial dynamics towards fission (19). Transcriptional upregulation of OPA1 occurred through NF-κB-responsive promoter elements, which maintained mitochondrial integrity (33). Moreover, previous studies proved that increased NF-κB activity could suppress NLRP1 inflammasomes in squamous cell carcinoma (34). The transcriptome sequencing of control and NLRP1-overexpressing PC-9 cells uncovered significant changes in NF-κB signaling in LUAD cells upon NLRP1 overexpression, but whether NLRP1 modulated mitochondrial dynamics through this pathway remained unanswered. Our data showed that the phosphorylation protein level of NF-κB (p-p65) was decreased in NLRP1-overexpression LUAD cells. Nevertheless, p-DRP1 inhibition and mitochondrial fission could be restored by NF-κB activator 1. Therefore, we concluded that NLRP1 overexpression might promote mitochondrial fusion by inhibiting NF-κB activation.

Cisplatin has been the most widely utilized chemotherapeutic agent in clinical practice, but it also causes lung damage by increasing oxidative stress (35). Long-term exposure to cisplatin could lead to the development of resistance, which was considered a significant contributor to disease recurrence and a major challenge in LUAD treatment (36). Cisplatin-induced interaction with mitochondria triggers apoptosis (37), whereas mitochondrial fission confers resistance to oxidative stress, leading to metastatic relapse (38). DRP1-dependent remodeling of mitochondrial morphology was reported to increase cisplatin resistance in nasopharyngeal carcinoma (39). Targeting lactate dehydrogenase-A enabled the elimination of cisplatin-resistant cells by increasing ROS levels (40). Our data showed that NLRP1 overexpression sensitized LUAD cells to cisplatin. Compared to cisplatin treatment alone, cisplatin combined with NLRP1 overexpression resulted in a more significant MMP loss. We also noted that NLRP1 might enhance cisplatin sensitivity of LUAD by inhibiting DRP1 phosphorylation, but further confirmation is needed.

Together, our study offered a novel insight on the involvement of mitochondrial dynamics in LUAD, which was influenced by the inflammasome-related protein NLRP1. We revealed that NLRP1 promoted mitochondrial fusion, and further suppressed tumor growth and enhanced cisplatin sensitivity, which was mediated by inhibition of NF-κB. We proposed that NLRP1 might be a novel target for therapeutic intervention of LUAD.

## Supplementary Material

Click to view [pdf].
