# Supplementary material for: NLRP1 inhibits lung adenocarcinoma growth through mediating mitochondrial dysregulation in an inflammasome-independent manner
Source: Braz J Med Biol Res. 2024 Sep 6;57:e13885. doi: 10.1590/1414-431X2024e13885 (PMC11379352; doi:10.1590/1414-431X2024e13885)
Supplement: Supplementary file 1 [file 1414-431X-bjmbr-57-e13885-suppl.pdf]

Figure S1. Correlation between NLRP1 and lung squamous cell carcinoma (LUSC). **A**, Expression level of NLRP1 in LUSC and adjacent non-tumor tissues from The Cancer Genome Atlas (TCGA) database. **B**, Associations of NLRP1 expression with TNM stage in LUSC. **C**, Prognostic value of NLRP1 in LUSC for disease-free survival (DFS) and overall survival (OS). All these data were analyzed using GEPIA2. T: Tumor tissue; N: Normal tissue. Data are reported as mean and SD. *P<0.05; Student's *t*-test.

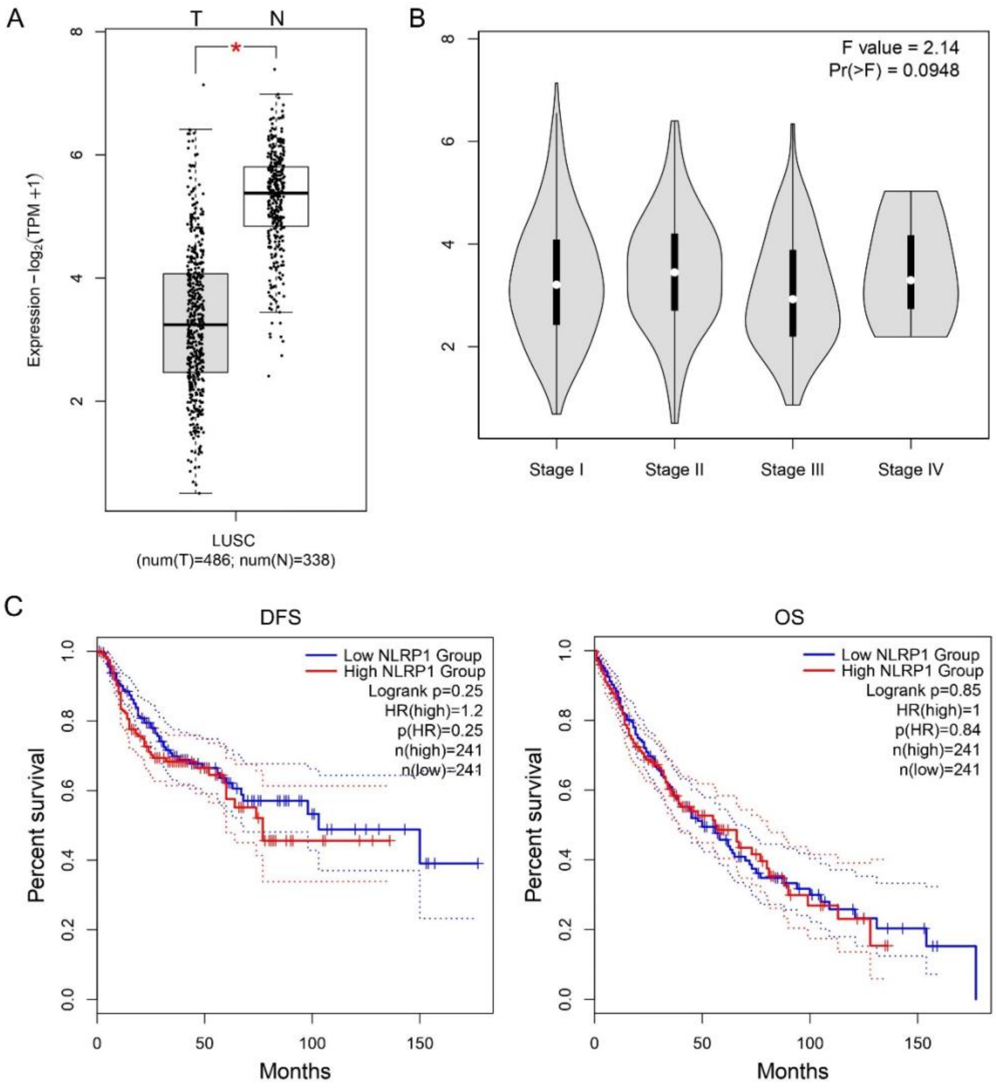

Figure S2. Detection of NLRP1 expression in lung cancer cell lines. **A**, Basal expression level of NLRP1 in human lung cancer cell lines. **B**, Western blot detection of NLRP1 expression in transfected cells. **C**, Cell viability of H1299 cells with NLRP1 knockdown. Data are reported as mean and SD. *P<0.05, **P<0.01; Student's *t*-test.

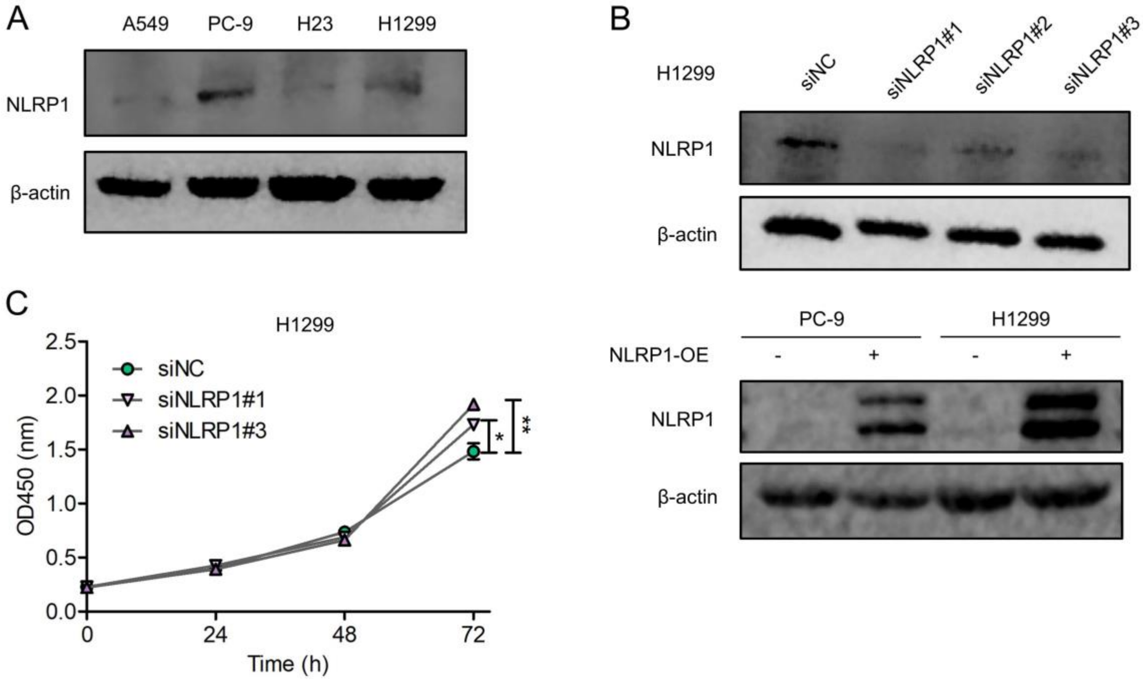

Table S1. Antibodies used in this research.

Antibodies	Source	Catalog number
Purified anti-NLRP1 antibody	Biolegend	679802
NLRP1 Rabbit Polyclonal antibody	Proteintech	12256-1-AP
PCNA (D3H8P) XP® Rabbit mAb	Cell Signaling Technology	13110
DRP1 (D6C7) Rabbit mAb	Cell Signaling Technology	8570
Phospho-DRP1 (Ser616) (D9A1) Rabbit mAb	Cell Signaling Technology	4494
DRP1 Polyclonal antibody	Immunoway	YT1414
Anti-Mitofusin 2 + Mitofusin 1 antibody [3C9]	Abcam	ab57602
Anti-Mitofusin 2 antibody [6A8]	Abcam	ab56889
OPA1 Polyclonal antibody	Proteintech	27733-1-AP
GSDMD N-terminal rabbit pAb	Immunoway	YT7991
GSDME N-terminal rabbit pAb	Immunoway	YT7990
β-Actin (8H10D10) Mouse mAb	Cell Signaling Technology	3700
IRDye® 800CW Goat anti-Rabbit IgG secondary antibody	LI-COR	C50331-05
IRDye® 680RD Goat anti-Mouse IgG secondary antibody	LI-COR	C50113-05
Anti-rabbit IgG (H+L), F(ab')2 Fragment (Alexa Fluor® 488 Conjugate)	Cell Signaling Technology	4412
